# Gold nanoparticles induced cloudy swelling to hydropic degeneration, cytoplasmic hyaline vacuolation, polymorphism, binucleation, karyopyknosis, karyolysis, karyorrhexis and necrosis in the liver

**DOI:** 10.1186/1476-511X-10-166

**Published:** 2011-09-22

**Authors:** Mohamed Anwar K Abdelhalim, Bashir M Jarrar

**Affiliations:** 1 Department of Physics and Astronomy, College of Science, King Saud University, Saudi Arabia; 2 College of Applied Medical Sciences, Al-Jouf University, P.O. Box 2014, Skaka - Al-Jouf, Saudi Arabia

**Keywords:** gold nanoparticles, size, hepatic tissue, histology, hydropic degeneration, nanotoxicity, rats

## Abstract

**Background:**

Nanoparticles (NPs) can potentially cause adverse effects on organ, tissue, cellular, subcellular and protein levels due to their unusual physicochemical properties. Advances in nanotechnology have identified promising candidates for many biological and biomedical applications. The aim of the present study was to investigate the particle-size, dose and exposure duration effects of gold nanoparticles (GNPs) on the hepatic tissue in an attempt to cover and understand the toxicity and their potential therapeutic and diagnostic use.

**Methods:**

A total of 70 healthy male Wistar-Kyoto rats were exposed to GNPs received 50 or 100 ul of GNPs infusion of size (10, 20 and 50 nm for 3 or 7 days) to investigate particle-size, dose and exposure duration effects of GNPs on the hepatic tissue.

**Results:**

In comparison with respective control rats, exposure to GNPs doses has produced alterations in the hepatocytes, portal triads and the sinusoids. The alterations in the hepatocytes were mainly vacuolar to hydropic degeneration, cytopasmic hyaline vacuolation, polymorphism, binucleation, karyopyknosis, karyolysis, karyorrhexis and necrosis.

**Conclusions:**

The hepatocytes swelling might be exhibited as a result of disturbances of membranes function that lead to massive influx of water and Na^+ ^due to GNPs effects accompanied by leakage of lysosomal hydrolytic enzymes that lead to cytoplasmic degeneration and macromolecular crowding. Hydropic degeneration is a result of ion and fluid homestasis that lead to an increase of intracellular water. The vacuolated swelling of the cytoplasm of the hepatocytes of the GNPs treated rats might indicate acute and subacute liver injury induced by the GNPs. Binucleation represents a consequence of cell injury and is a sort of chromosomes hyperplasia which is usually seen in regenerating cells. The induced histological alterations might be an indication of injured hepatocytes due to GNPs toxicity that became unable to deal with the accumulated residues resulting from metabolic and structural disturbances caused by these NPs. These alterations were size-dependent with smaller ones induced the most effects and related with time exposure of GNPs. The appearance of hepatocytes cytoplasmic degeneration and nuclear destruction may suggest that GNPs interact with proteins and enzymes of the hepatic tissue interfering with the antioxidant defense mechanism and leading to reactive oxygen species (ROS) generation which in turn may induce stress in the hepatocytes to undergo atrophy and necrosis. More histomorphologcal, histochemical and ultrastrucural investigations are needed in relation of the application of GNPs with their potential role as a therapeutic and diagnostic tool.

## Introduction

The rats exposed to aerosols of GNPs revealed that the NPs were rapidly taken into the system with the highest accumulation in the lungs, aorta, esophagus and olfactory bulb [1]. Moreover, NPs are believed to be more biologically reactive than their bulk counter parts due to their small size and larger surface area to volume ratio [1,2].

Although some scientists consider NPs as nontoxic, there are other studies reporting the toxic effects of NPs [3-5]. Although some NPs may appear to be nontoxic, other cellular mechanisms such as cell signaling and other normal cellular functions may be disrupted and are currently undergoing further investigation [6,7]. The toxicity of NPs is being addressed by number of standardized approaches with in vitro, in vivo as well as detailed genomic or biodistribution studies [7]. It has been shown that NPs may produce in vitro toxicity in some cell-based assays, but not in others. This may be a result of interference with the chemical probes, differences in the innate response of particular cell types, or other factors [8]. In addition, GNPs are used as carriers for the delivery of drugs and genes [9].

Gold in its bulk form has long been considered an inert, noble metal with some therapeutic and even medicinal value hence GNPs are thought also to be relatively non-cytotoxic [10]. Yet there are differing reports of the extent of the toxic nature of these particles owing to the different modifications of the GNPs, surface functional attachments and shape and diameter size of the nanospheres [11,12]. Moreover, the metallic nature of the metal derived NPs and the presence of transition metals encourages the production of reactive oxygen species (ROS) leading to oxidative stress [13,14].

The histological and the histochemical characterization in the hepatic tissues due to GNPs are not documented and have not yet been identified. In the present study, an attempt has been made to characterize the possible histological alterations in the hepatic tissues following experimental GNPs and, if so, whether are related to the size of these NPs and the time of exposure.

The present study was carried out to investigate the particle-size, dose and exposure duration of GNPs on the hepatic tissue in an attempt to cover and understand the toxicity and their potential therapeutic and diagnostic use in relation with the time of exposure.

## Materials and methods

A total of 70 healthy male Wistar-Kyoto rats obtained from the Laboratory Animal Center (College of Pharmacy, King Saud University, Saudi Arabia). The rats nearly of the same age (12 weeks old) and weighing 220-240 gm of King Saud University colony were used. Animals were randomly divided into groups, 12 GNPs-treated rats groups and one control group (NG). Following a period of stabilization (7 days), 10, 20 and 50 nm GNPs were administered intraperitonealy at the rate for 3 or 7 days as follows: Group 1: received infusion of 50 μl GNPs of size 10 nm for 3 or 7 days (n = 10); Group 2: received infusion of 50 μl GNPs of size 20 nm for 3 or 7 days (n = 10); Group 3: received infusion of 50 μl GNPs of size 50 nm for 3 or 7 days (n = 10); Group 4: received infusion of 100 μl GNPs of size 10 nm for 3 or 7 days; (n = 10); Group 5: received infusion of 100 μl GNPs of size 20 nm for 3 or 7 days (n = 10); Group 6: received infusion of 100 μl GNPs of size 50 nm for 3 or 7 days; (n = 10); Control group: received no gold nanoparticles (n = 10).

The rats were maintained on standard laboratory rodent diet pellets and were housed in humidity and temperature-controlled ventilated cages on a 12 h day/night cycle. Two animals from each group were killed by dislocation of the neck at intervals of 3 and 7 days of treatment with GNPs. All experiments were conducted in accordance with the guidelines approved by King Saud University Local Animal Care and Use Committee.

Fresh portions of the lateral lobes of the liver from each rat were cut rapidly, fixed in neutral buffered formalin (10%), then dehydrated, with grades of ethanol (70, 80, 90, 95 and 100%). Dehydration was then followed by clearing the samples in 2 changes of xylene. Samples were then impregnated with 2 changes of molten paraffin wax, then embedded and blocked out. Paraffin sections (4-5 um) were stained with hematoxylin and eosin the conventional histological and stain according to Pearse [15]. Stained sections of control and treated rats were examined for alterations in the architecture, portal triads, hepatocytes, sinusoids and for the presence of degeneration, necrosis, fatty change and portal fibrosis.

## Results and Discussions

The 10 and 20 nm GNPs show spherical shape while the 50 nm GNPs show hexagonal shape. The mean size for GNPs was calculated from the images taken by the transmission electron microscope (TEM). The 10 nm GNPs was of mean size 9.45 ± 1.33 nm, 20 nm GNPs was of mean size 20.18 ± 1.80 and the 50 nm GNPs was of mean size 50.73 ± 3.58 [16].

### Histological alterations

**Figure 1:**
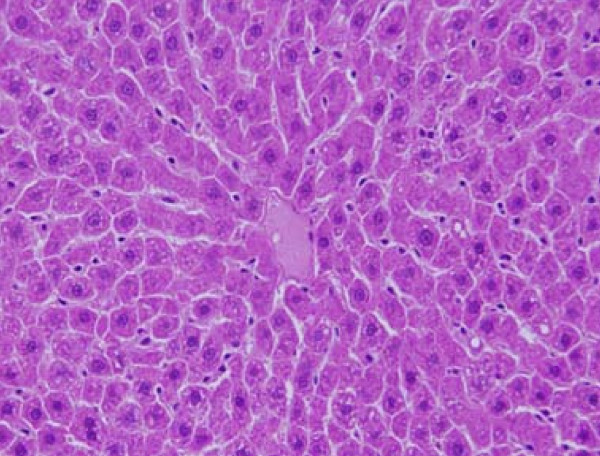
**GNPs-normal rat demonstrating normal hepatocytes**.

GNPs-normal rat demonstrating normal hepatocytes are shown in Figure 1. In comparison with the control group, the following histological alterations were detected in the liver of GNPs treated rats.

### Cloudy swelling

**Figure 2:**
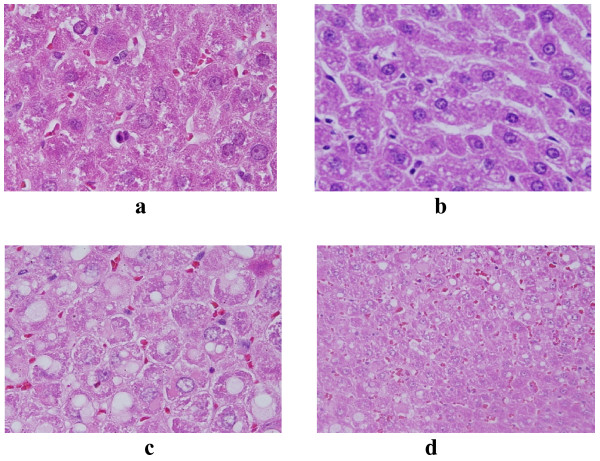
**GNPs-treated rat**. (A) GNPs-treated rat received 50 μl of 10 nm particles for 3 days demonstrating hepatocytes cloudy swelling. (B) GNPs-treated rat received 100 μl of 10 nm particles for 7 days demonstrating hydropic degeneration. (C) GNPs-treated rat received 50 μl of 10 nm particles for 3 days demonstrating hyaline inclusions. (D) GNPs-treated rat received 100 μl of 10 nm particles for 7 days demonstrating hyaline vacuolation.

hepatocytes exhibited cloudy swelling with pale cytoplasm and poorly delineated and displaced nuclei in all GNPs treated rats. This ballooning degeneration was more prominent with 100 μl dose than 50 μl one and with 10 nm size particles than the larger ones (Figure 2a). This swelling might be exhibited as a result of disturbances of membranes function that lead to massive influx of water and Na^+ ^due to GNPS effects. Cellular swelling might be accompanied by leakage of lysosomal hydrolytic enzymes that lead to cytoplasmic degeneration and macromolecular crowding [17].

### Hydropic degeneration

vacuolization of the hepatocytes cytoplasm was seen and increased in severity in the liver of rats received 100 μl of 10 nm GNPs with less vacuolar degeneration with larger ones. More vacuolar degeneration was observed in the hepatocytes of rats exposed to 7 days than ones exposed to 3 days (Figure 2b). Hydropic degeneration is a result of ion and fluid homestasis that lead to an increase of intracellular water [18]. The vacuolated swelling of the cytoplasm of the hepatocytes of the GNPs treated rats might indicate acute and subacute liver injury induced by these NPs.

### Hyaline inclusions and hayaline vacuolation

inclusions similar to Mallory hyaline bodies and hayaline vacuolations were detected in the cytoplasm of some hepatocytes of rats received 100 μl of 10 nm GNPs. This alteration was less prominent in rats exposed to larger particles (Figures 2c and 2d).

### Nuclear polymorphism

variable nuclei sizes were observed in some hepatocytes. This change became apparent after 7 days of 50 nm GNPs administration. Some studies indicate that nuclear polymorphism is seen in hepatic dysplasia and carcinomatous lesion [19].

### Karyopyknosis

**Figure 3:**
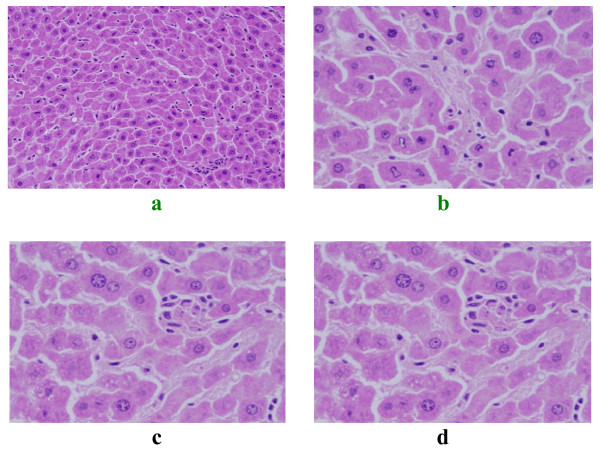
**GNPs-treated rat**. (A) GNPs-treated rat received 100 μl of 50 nm particles for 3 days demonstrating pyknotic hepatocytes. (B) GNPs-treated rat received 100 μl of 50 nm particles for 3 days demonstrating clumping and condensation of the chromatin materials in the periphery of the nuclei together with irregularity nuclear membranes. (C) GNPs-treated rat received 50 μl of 50 nm particles for 7 days demonstrating karyorrhexis. (D) GNPs-treated rat received 100 μl of 50 nm particles for 7 days demonstrating karyolysis.

pyknotic nuclei were seen in some hepatocytes of GNPs treated rats. Some pyknotic hepatocytes of rats received 100 μl of 50 nm size particles exhibited clumping and condensation of the chromatin materials in the periphery of the nuclei together with irregularity nuclear membranes (Figures 3a and 3b). Karyopyknosis ia an irreversible condensation of chromatin in the nucleus of a cell undergoing necrosis or apoptosis [20].

### Karyorrhexis

some hepatocytes of rats received 50 nm GNPs showed nucleoli disappearance (Figure 3c). This nuclear damage was more prominent after 7 days of exposure to NPs. Karyorrhexis is a sort of destructive fragmentation of the nucleus proceeded by pyknosis and is followed by karyolysis [21].

### Karyolysis

this alteration appeared mainly in the liver of GNPS-treated rats exposed to 100 ul of 50 nm size particles (Figure 3d). Karyolysis is the complete dissolution of the chromatin matter of a dying cell [22].

### Binucleation

**Figure 4:**
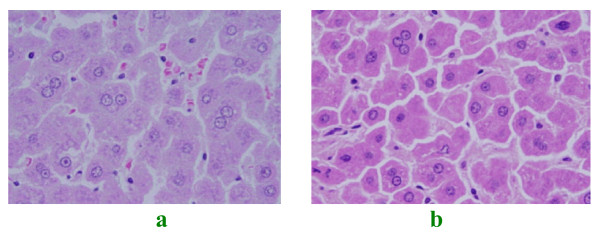
**GNPs-treated rat**. (A) GNPs-treated rat received 100 μl of 50 nm particles for 3 days demonstrating binucleation. (B) GNPs-treated rat received 100 μl of 20 nm particles for 7 days demonstrating binucleation.

occasional binucleation and to lesser extent polynucleation were observed in GNPs treated rats. This change was more prominent in rats exposed to 100 μl of 50 nm size GNPs (Figures 4a and 4b). Binucleation represents a consequence of cell injury and is a sort of chromosomes hyperplasia which is usually seen in regenerating cells [23].

### Necrosis

**Figure 5:**
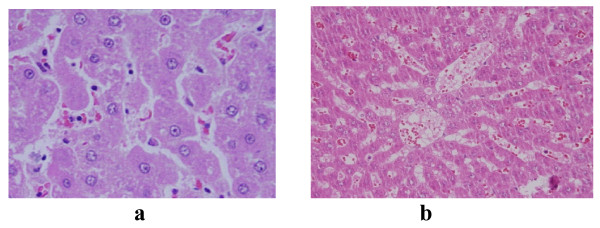
**GNPs-treated rat**. (A) GNPs-treated rat received 50 μl of 10 nm particles for 3 days demonstrating necrotic hepatocytes. (B) GNPs-treated rat received 50 μl of 10 nm particles for 3 days demonstrating hepatic sinusoidal dilatation.

sporadic spotty well-defined necrosis was noticed in some hepatocytes of GNPs treated rats (Figure 5a). The insulted cells exhibited highly eosinophilic amorphus cytoplasm with occasional apoptotic characterization (Figure 3l). This alteration was detected in the liver of rats exposed to 10 nm size particles and to lesser extent with 20 nm particles but was not seen with those exposed to 50 nm size particles. Apoptic alteration might be followed organelles swelling specially mitochondria, endoplasmic reticulum and rupture of lysosomes which might lead to amorphous eosinophilic cytoplasm as an initial sign in the sequence of hepatocytes necrosis before shrinking and dissolution of nulei [24]. The seen hepatocytes necrosis due to GNPs exposure might indicate oxidative stress on these cells by glutathione depletion.

### Hepatic sinusoids dilatation

hepatic sinusoids were more dilated in rats received 50 μl of 10 nm GNPs than those exposed to larger particles (Figure 5b). This alteration was almost the same among rat exposed to GNPs for 3 or 7 days. This vascular alteration is characterized by focal dilatation of sinusoidal spaces associated hepatocytes atrophy and necrosis [25].

Abdelhalim and Bashir, 2011 have reported that GNPs-treated rat received 100 μl of 10 nm particles for 7 days demonstrating apoptotic characterization, GNPs-treated rat received 100 μl of 10 nm particles for 3 days demonstrating inflammatory cell infiltration, GNPs-treated rat received 50 μl of 10 nm particles for 7 days demonstrating Kupffer cells hyperplasia, GNPs-treated rat received 100 μl of 10 nm particles for 7 days demonstrating hepatic fatty degeneration and GNPs-treated rat received 50 μl of 20 nm particles for 7 days demonstrating hepatic central vein intima disruption [16].

None of the above alterations were observed in the liver of any member of the control group (Figure 1).

## Conclusions

Histological alterations by GNPs exposure as shown in the results of the present work could be an indication of injured hepatocytes due to GNPs toxicity that become unable to deal with the accumulated residues resulting from metabolic and structural disturbances caused by these particles. One might conclude that these alterations are size-dependent with smaller ones induced more damage with relation with the time exposure of GNPs.

The appearance of hepatocytes cytoplasmic degeneration and nuclear destruction may suggest that GNPs interact with proteins and enzymes of the hepatic tissue interfering with the antioxidant defense mechanism and leading to reactive oxygen species (ROS) generation which in turn may induce stress in the hepatocytes to undergo atrophy and necrosis.

More histomorphological, histochemical and ultrastructural investigations are needed to correlate the biomedical application of GNPs with the potential threat of their therapeutic and diagnostic use.

## Competing interests

The authors declare that they have no competing interests.

## Authors' contributions

MAKA and BMJ have analyzed data, interpreted and written the final draft of this manuscript. The animal model used in this study was obtained from the Laboratory Animal Center (College of Pharmacy, King Saud University, Saudi Arabia). MAKA has conceived the study and its design and obtained research grants for this study. Moreover, both authors have read and approved the final manuscript.
